# Ultrasound-Assisted Urea-Water Solution (AdBlue) Droplets Vaporization: A Mathematical Model for Film and Volumetric Regimes with Implications in NOx Emission Control

**DOI:** 10.3390/mi16090996

**Published:** 2025-08-29

**Authors:** Claudiu Marian Picus, Ioan Mihai, Cornel Suciu

**Affiliations:** Faculty of Mechanical Engineering, Automotive and Robotics, Stefan cel Mare University, 720229 Suceava, Romania; claudiu.picus@usm.ro

**Keywords:** ultrasonic excitation, AdBlue, atomization, SCR, mathematical model, NOx

## Abstract

The vaporization of urea–water solution (AdBlue) plays a critical role in the performance of selective catalytic reduction (SCR) systems for modern diesel engines. This study presents mathematical models describing the vaporization of AdBlue droplets under ultrasonic excitation generated by a magnetostrictive effect, focusing on both film and volumetric regimes. The models rigorously incorporate heat and mass transfer equations, including acoustic cavitation effects induced by ultrasound. The influence of magnetostrictive-induced atomization and combined inductive preheating on droplet detachment and SCR catalyst efficiency was analyzed. Additionally, the impact of ultrasound frequency and amplitude on thermal vaporization efficiency and reactive mixture formation was investigated with the aim of enhancing NOx emission reduction. Model validation against literature data confirmed the practical applicability of the proposed approach, offering valuable insights for optimizing ultrasound-assisted AdBlue injection systems.

## 1. Introduction

Diesel engines are a major source of emissions of nitrogen oxides (NOx), compounds with significant undesirable impact on human health and the environment. In this context, selective catalytic reduction (SCR) technology has been widely adopted in diesel engines and in industry as an efficient solution for the conversion of NOx to molecular nitrogen and water vapor by injecting an aqueous solution of urea (AdBlue) into the exhaust gas [1,2,3,4].

In Europe, the introduction of on-road Real Driving Emissions (RDE) testing and the progressive tightening of Euro 6/VI requirements have been central to reducing NOx from vehicles in real use [2]. At the same time, official EU indicators document long-term declines in transport air-pollutant emissions, reflecting successive standards and in-use controls [3]. Recent EU testing programs further show improved emissions performance of modern light-duty vehicles across laboratory and on-road conditions [5], while large-scale remote-sensing campaigns reveal the real-world distribution of heavy-duty vehicle emissions in current fleets [6]. These EU data motivate accurate modeling of AdBlue droplet vaporization under conditions representative of real operation.

Previous studies [1] showed that the conversion efficiency is directly dependent on the degree of atomization and the vaporization rate of the AdBlue solution, factors that influence the reactive contact between urea and exhaust gases. Conventional injection systems have significant limitations in terms of AdBlue droplet size (30–100 μm) and distribution, which can lead to the formation of urea deposits in the form of solid salts inside the SCR catalyst microchannels with a direct effect on efficiency reduction.

The conducted studies have highlighted the potential of using magnetostrictive effect as a method to intensify atomization processes with a direct result on increasing the efficiency of the vaporization process. The use of the magnetostrictive effect led [1] to obtaining microdroplets with diameters ranging from 3 to 10 μm. The action of the ultrasonic field specific to the magnetostrictive effect causes the occurrence of the acoustic cavitation phenomenon, which favors the fragmentation of droplets, thus increasing the evaporation surface area and the homogenization of the reactive mixture.

The model proposed in this paper provides a unified approach to the two main regimes of the vaporization process—pellicular and volumetric—integrating ultrasound effects in each phase of heat and mass transfer. Special attention is paid to how acoustic cavitation influences both evaporation at the droplet surface and internal processes, thus contributing to a more accurate description of the physicochemical behavior of the AdBlue solution under real operating conditions.

A brief description of the research method and the operating assumptions used in the presented modeling is provided in Section 2.1.

In selective catalytic reduction (SCR) systems [7], efficient heat transfer control is a key determinant for maintaining an optimal catalyst temperature, which is essential for the complete unfolding of the chemical reactions for the conversion of nitrogen oxides. Heat transfer in this context is the result of a complex interplay between several mechanisms—convective, conductive, radiative, and diffusive—each contributing in its own particular way to the thermal dynamics of the system. These processes involve the uptake of thermal energy from flue gases or external sources and its redistribution to the areas of interest: either in the catalyst mass (volume regime) or to structural elements (surface regime).

Of particular importance is the diameter of the injected AdBlue droplets. Majewski [4] points out that dosing systems must ensure the most accurate injection of urea to ensure the effectiveness of SCR reactions and uniform mixing of urea and ammonia with the exhaust gas. A better atomization of the injected urea droplets (smaller droplet size) allows a more complete conversion to ammonia and can minimize the risk of fouling with solid deposits. Urea injectors produce droplets of 30–100 µm, expressed as Sauter mean diameter (SMD). Droplets below 10 µm can be useful to minimize deposits in SCR systems designed to provide sustained NOx reduction at temperatures up to 423–473 K.

Ponnathpur, C. et al. [8], patented an ultrasonic vaporizer, which is capable of producing diesel droplets of 5–7 µm. According to Rajan and Pandit [9], the frequency of ultrasonic vibration determines the droplet size in ultrasonic droplet atomization, allowing the production of very small droplets (1–5 μm). The thickness of the film formed on the vibrating surface also has an effect on the droplet size, which can be controlled by adjusting the liquid flow rate.

The thermodynamic transformations of the AdBlue solution inside the SCR system in the NOx conversion process differ depending on the working area.

- When the AdBlue solution is injected onto the hot surfaces of the exhaust system, a phase transformation occurs after reaching saturation temperature, i.e., vaporization. If the surface temperature is too low (below the vaporization temperature) only partial phase transformation occurs. If the surface temperature is much above the saturation temperature, pseudo-sublimation occurs when AdBlue is injected onto the surface.
- In the case of AdBlue micro-droplets entrained in motion by the hot flue gas stream, vaporization proceeds convectively in volume, in which case the phenomena are characterized by mass transport and enhanced thermal diffusion.
- Following the injection of the AdBlue solution, the liquid droplets undergo a biphasic transformation through a continuous mass transfer cycle until they enter the SCR honeycomb.

This study developed and validated a unified mathematical model for ultrasound-assisted AdBlue vaporization in both film and volumetric regimes, explicitly incorporating heat transfer, diffusion, and acoustic cavitation. The goal was to provide a predictive framework for optimizing SCR systems under real operating conditions, with implications for improved NOx reduction efficiency.

## 2. Methodology and Mathematical Framework

### 2.1. Research Method: Thermal Operating Context and Assumptions

This subsection summarizes the operating-temperature context and the modeling assumptions that frame our analysis. These elements are integral to the research method adopted for the subsequent derivations and simulations.

Proper management of these phenomena is essential not only to optimize chemical conversion but also to prevent adverse effects, such as urea crystallization or solid deposit formation, which can compromise the performance and durability of the SCR system [8]. Integrating a coherent thermal model in the analysis of ultrasonically assisted AdBlue behavior allows a more accurate assessment of overall system efficiency and supports thermal optimization. The gas temperature at the end of the exhaust process, in the cycle of a fast-burning diesel engine, is typically 700–800 K [10]. Upstream of the SCR catalyst, typical temperatures are ≈400–800 K as heat is lost along the exhaust line [11]. Prior studies indicate an effective temperature window for urea decomposition of roughly 453–723 K [12,13,14,15].

Therefore, across varying engine operating conditions, adequate heat transfer must be ensured to maintain the catalyst honeycomb near its optimum temperature. Particular attention is required during cold operation, when exhaust temperatures may be too low to reach the catalyst’s optimal range. In the AdBlue injection zone and the SCR catalyst, understanding how heat transfer and physico–chemical reactions interact is essential to achieving efficient NOx reduction in diesel exhaust.

### 2.2. Ultrasonic Device and Operating-Frequency Treatment

Ultrasonic excitation is provided by a magnetostrictive actuator mechanically coupled to the injector region to generate an acoustic field in the vicinity of the injection zone and within the gas volume. The generator controls the driving frequency *f*_US_ and the displacement amplitude *A*_US_, which govern atomization and cavitation.

For the device used in this study, the magnetostrictive transducer operates in the fundamental band 20–40 kHz. Increasing *f*_US_ generally reduces the mean droplet diameter, consistent with classical ultrasonic-atomization correlations [9].

Prior experiments of the authors reported microdroplets in the 3–10 μm range for magnetostrictive atomization, whereas conventional injectors typically yield 30–100 μm (SMD) [1]. In the present model, the influence of *f*_US_ and *A*_US_ enters through (i) the initial droplet-size distribution chosen for each case and (ii) the interfacial transfer coefficients that increase with finer atomization. This is consistent with the known frequency–droplet-size dependence [9]. For reproducibility, the device’s operating band (frequency range, amplitude, electrical power, coupling, and geometry) can be reported as available; the conclusions of the study do not hinge on a specific numeric setting.

### 2.3. Convective Vaporization of AdBlue Droplets Evolving in a Hot Gaseous Medium with or Without Calefaction

In order to rigorously reveal how the efficiency of SCR systems is influenced by the injection of AdBlue solution, a detailed analysis of the thermal interaction between aqueous urea droplets and the exhaust gas under varying temperatures and different flow regimes is required. The study considers three key aspects:

- **Vapour formation** is when AdBlue droplets are injected onto a surface subject to a magnetostrictive effect; a phenomenon known as calefaction may occur if atomized droplets detached from the surface move into a volume of hot gas. The phenomenon must take place in the immediate vicinity of the solid surface without intimate contact with it, and the droplets are agitated by ultrasonic waves obtained by the magnetostrictive effect. Thermal diffusion can also be taken into account in the calculations. This regime involves a complex volume interaction, where the velocity and temperature of the gaseous medium directly influence the evaporation process.
- **Evaporation on contact with hot surfaces** is a scenario that simulates direct injection on hot components of the exhaust manifold or catalyst structure when the droplet jet hits the walls of the exhaust duct of the diesel engine. In this case, the heat transfer is conductive and radiative and takes a significant part in vaporization.
- **The mechanism of formation of solid deposits** is directly related to the operating temperature and is characterized by the appearance of solid deposits of AdBlue salts either on the injector nozzle or on the front surface of the catalyst nozzle. This phenomenon is mainly caused by high temperatures, temperature inhomogeneities, and the unfinished evaporation process of the AdBlue droplets.

The mathematical modeling adopted in this study allows the consideration of variable quantities such as droplet diameter, droplet local temperature, ambient temperature, exhaust gas temperature, and the density of the phases involved. The heat transfer process that takes place between the AdBlue droplet and the flue gas is governed by complex thermo-gas-dynamic phenomena, specific to two-phase flowing systems.

These phenomena differ fundamentally from classical situations, in which the phases are either mixed (liquid–vapor) or completely separated. In modeling practice, the exact delineation of the boundaries between these two states is difficult to achieve, requiring an adaptive and robust approach to the transfer equations.

The determination of the thermophysical parameters of a droplet moving in a gaseous medium with a known velocity requires taking into account the interfacial heat exchange and the mechanical coupling effects between phases. The aim is to quantify the influence of the exhaust gas velocity on the evolution of temperature and droplet diameter in order to optimize the vaporization process and reduce the risk of solid deposition in the SCR system.

**Figure 1:**
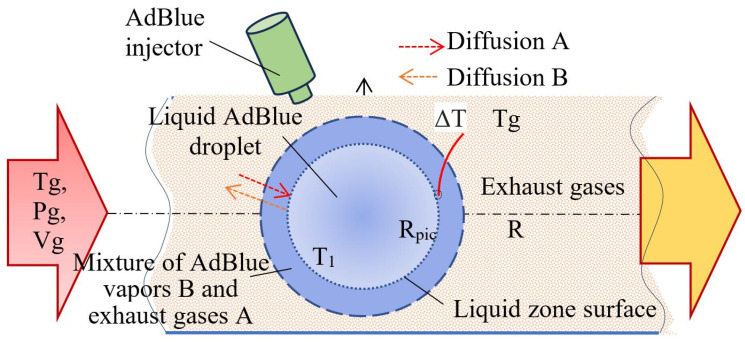
Thermophysical processes of a liquid droplet vaporizing in a gaseous medium.

For very small liquid droplets, it can be assumed that their velocity is approximately equal to that of the gas phase. The flow of a two-phase mixture [16,17,18] is governed by the interphase heat transfer, mechanical frictional work between the liquid and vapor phases and their frictional work with the gaseous medium, the continuity equation, and the law of conservation of energy. Figure 1 schematically considers the exhaust manifold of a diesel engine including the thermophysical processes of a liquid droplet evaporating in a gaseous medium.

The parameters of the biphasic medium consisting of AdBlue droplets evaporating as they move and the isothermal gaseous medium are considered. It can be seen that the exhaust gas enters the injection zone with initial temperature *T_g_*, pressure *P_g_*, and volume *V_g_*. Upon contact of an AdBlue droplet with the hot gases, a film layer of AdBlue vapor mixture of molecular weight B is formed at the droplet–gas interface, which interacts with the flue gas A by diffusion. The diffusive exchange between the two substances occurs bi-directionally, both from the liquid droplet to the flue gas and vice versa (red arrows). Since convective heat exchange takes place between the gas and the liquid phase, it is of interest to determine the convection coefficient *α* [W/m^2^ K] with which the heat flux and the amount of heat exchanged can be determined. To solve this, one resorts to similarity theory, in which case the Nusselt criterion for liquid droplets in a hot gaseous medium has the following relation (1):

(1) $$N_{u}=\;2+0.6{\cdot Re}^{0.5}\cdot {Pr}^{0.333}.$$

Knowing that the specific form of the Nusselt criterion is the following:

(2) $$N_{u}=\frac{\alpha {\cdot d}_{o}}{\lambda_{f}}.$$

Then, by equating relations (1) and (2) the following results:

(3) $$\alpha_{c}=\frac{\lambda_{g}}{d_{pAD}}\left[2+0.6{Re}^{0.5}\cdot {Pr}^{0.333}\right],$$

where *α_c_* [W/m^2^ K]—the convective heat coefficient, *d_pAD_* [m]—the characteristic dimension which, as a rule, is taken equal to the diameter of the AdBlue droplet, *Re*—the Reynolds number, *Pr*—the Prandtl invariant. Taking into account the expression of the invariant *Pr*:

(4) $$\mathrm{Pr}=\frac{v}{a}=\frac{v\cdot \rho \cdot Cp}{\lambda }=\frac{\eta \cdot Cp}{\lambda },$$

the convection coefficient *α_c_* is obtained as follows:

(5) $$\alpha_{c}=\frac{\lambda_{g}}{d_{pAD}}\left[2+0.6{\left(\frac{u_{AD}\cdot d_{pAD}\cdot \rho_{AD}}{\mu_{AD}}\right)}^{0.5}\cdot {\left(\frac{\mu_{AD}\cdot Cp_{AD}}{\lambda_{AD}}\right)}^{0.333}\right],$$

where *λ_g_* [J/kgK]—the thermal conduction coefficient, *ρ_AD_* [kg/m^3^]—the density of the AdBlue solution, *Cp_AD_* [J/kgK]—the specific heat at constant pressure, *μ_AD_* [Pa·s]—dynamic viscosity of the AdBlue solution, $\lambda_{AD}$—the thermal conduction coefficient of AdBlue.

By analyzing the expression of the heat convection coefficient it can be observed that it varies inversely proportional to the diameter of the AdBlue droplets and directly proportional to their displacement velocity. It can be concluded that in order to enhance the connective heat exchange, it is necessary to atomize the AdBlue droplets as finely as possible and to find ways to increase the velocity of the liquid droplets. The duration of the AdBlue droplet injection process is very short, being in the order of 10 ÷ 20·10^−3^ s, in order to save solution and to avoid injecting large quantities that would lead to NOx increase by a mechanism that will be described later. According to theoretical calculations, the convective coefficient *α_c_* has very large values in the initial pulverization phase, and there is a lag of heat exchange with respect to mass exchange. Therefore, it is preferred to adopt an average value of convective heat exchange in the analytical calculations. The heat exchanged by convection during a *dτ* time, corresponding to a *dx* length traveled by the AdBlue particles with outer surface area *A_PAD_*, will be deduced from the following balance relation:

(6) $$\alpha_{c}A_{PAD}\left(T_{PAD}-T_{g}\right)d\tau ={\dot{m}}_{AD}Cp_{AD}dT_{PAD},$$

where *α_c_* [W/m^2^ K]—the convection coefficient, *A_PAD_* [m^2^]—the area of spherical AdBlue droplets, *T_g_* [K]—temperature of the exhaust gas, *T_PAD_* [K]—the temperature of AdBlue droplets, *τ* [s]—time, ${\dot{m}}_{AD}$ [kg/s]—the mass flow rate of injected AdBlue solution, *Cp_AD_* [J/kgK]—the specific heat at constant pressure. The variation of the liquid phase temperature with time due to interface heat exchange is based on the following relation:

(7) $$u_{AD}\frac{dT_{PAD}}{dx}=-\frac{\alpha_{c}A_{PAD}\left(T_{g}-T_{PAD}\right)}{{\dot{m}}_{AD}Cp_{AD}}.$$

By substituting the expression of the convection coefficient *α_c_* into Equation (7), the following is obtained:

(8) $$u_{AD}\frac{dT_{PAD}}{dx}=-\frac{6\lambda_{g}}{{\rho_{AD}\cdot Cp}_{AD}\cdot d_{pAD}^{2}}\left(2+0.6{Re}^{0.5}\cdot {Pr}^{0.333}\right)\left(T_{g}-T_{PAD}\right).$$

If part of Equation (8) is substituted by the term a, as given in (9):

(9) $$a=\frac{6\lambda_{g}}{{\rho_{AD}\cdot Cp}_{AD}\cdot d_{pAD}^{2}}\left(2+0.6{Re}^{0.5}\cdot {Pr}^{0.333}\right),$$

the differential Equation (8) becomes as follows:

(10) $$u_{AD}\frac{dT_{AD}}{dx}=a\left(T_{g}-T_{PAD}\right).$$

If the substitution *u = T_g_ − T_PAD_*, is used and by integrating differential Equation (10), the following results:

(11) $$T_{l}=T_{g}-k_{1}e^{\frac{a}{u_{AD}}x}.$$

Knowing that *x* is the instantaneous path traveled by the drop, the constant *k*_1_ is determined by putting boundary conditions. After performing the calculations, the solution is obtained as follows:

(12) $$T_{l}=T_{g}-\left(T_{g}-T_{PAD}\right)\cdot \exp\left(-\frac{6\lambda_{g}\left(2+0.6Re^{0.5}P_{r}^{0.333}\right)}{\rho_{AD}CP_{AD}d_{pAD}^{2}}x\right).$$

**Figure 2:**
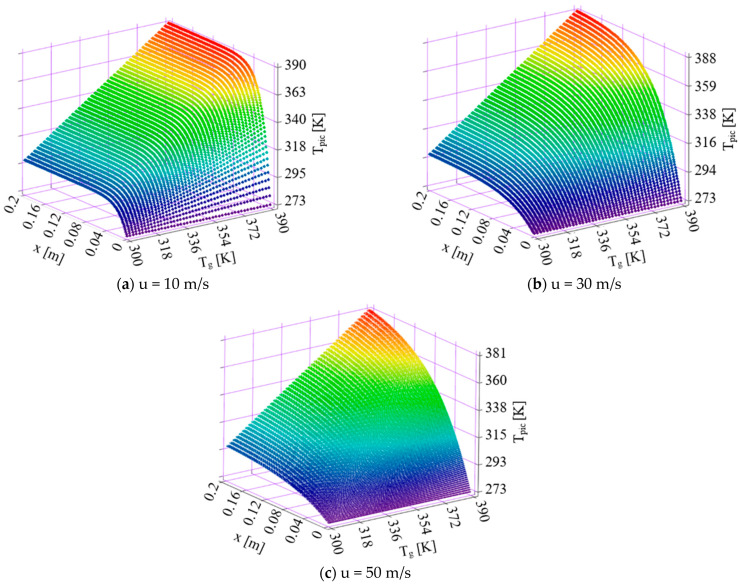
Variation of AdBlue droplet temperature with space traveled and exhaust gas temperature at different travel speeds.

Equation (12) was introduced in a MathCad14.0 environment for a 50 μm diameter Adblue droplet and allows the calculation of the liquid droplet temperature as a function of the space traveled through a high temperature biphasic gaseous medium, without taking into account the thermal diffusion phenomenon. During the cold start of a diesel engine, a high temperature can be achieved by the inductive exhaust gas preheating method. Using the results of analytically obtained calculations, the representations in Figure 2 were plotted.

The graphs given in Figure 2 show the variation of droplet temperature *T_pic_* [K] with exhaust gas temperatures *T_g_* [K] between 300–390 K and the space traveled by the AdBlue jet denoted in the figure by *x* reaching 0.2 m. The calculations were performed for different AdBlue droplet jet velocities of 10, 30, 50 m/s.

Program description (duration, outputs, and error control). The calculations were carried out in Mathcad14.0 for both volumetric (gas-volume) and pellicular (surface/film) regimes, consistent with the models presented in Section 2 and Section 3. For volumetric cases, trajectories were advanced from t = 0 to complete vaporization, and the resulting evaporation times *t*_evap_ are discussed in Section 5 (Results and Discussion). For the near-surface analysis illustrated in Figure 2, droplet heating and kinematics were evaluated along the flight path up to *x* = 0.2 m for jet velocities of 10, 30, and 50 m/s, with the time/distance evolutions of droplet diameter and temperature generated for the representative conditions shown there. To improve comparability with literature using different initial diameters, the subsequent discussion (Section 5) uses the D^2^-law normalization *t*/*d*_0_^2^. As an internal consistency check, paired analytical calculations with and without the thermal-diffusion term (Section 3) were performed, and calculations were carried out across gas temperature and travel speed.

By analyzing the obtained results, it was found that the temperature of AdBlue droplets traversing a length of 0.2 m is strongly dependent on the temperature of the medium in which vaporization takes place, the initial size of the liquid droplets, the space traveled, and the instantaneous velocity of the liquid droplets.

Two situations occur. The first situation occurs when the exhaust gases are cold and do not reach the optimum operating temperature of the SCR. This case occurs from cold engine start until temperatures close until the saturation values of the AdBlue solution are reached. The AdBlue droplets can be judged to approach saturation temperature and reach saturation temperature if the exhaust gas temperature is higher by 10–15 K. The second situation is when the AdBlue droplets are injected into the exhaust manifold in a gaseous medium at temperatures much higher than the saturation temperature.

From Figure 2, it can be observed that the saturation temperature (marked in red), which corresponds to vaporization, is reached at different values of the operating parameters. Although the properties of the AdBlue solution suggest a saturation temperature of 373.15 K, the calculations indicate that slight exceedance of this value may occur. This deviation can be attributed to the pressure exerted by the exhaust flue gas column.

It is known that for supercharged diesel engines [10], the exhaust stack pressure *p_r_* can reach higher values than atmospheric pressure since *p_r_* = (0.75… 0.98)*p_s_*, where *p_s_* is the supercharging pressure.

The adopted calculation model makes it possible to determine the vaporization behavior of the AdBlue droplets injected into the volume of hot diesel engine combustion gases around the saturation temperature, a phenomenon of interest when studying the deposition of AdBlue salts on the catalyst honeycomb. From the graphs shown in Figure 2, it can be seen that the higher the AdBlue droplet velocity, the faster the AdBlue droplet temperature increases.

## 3. Determination of the Diameter of the AdBlue Droplets Injected into the Exhaust Gas Volume of the SCR, Considering the Diffusion Phenomenon

In the analysis of the vaporization process of AdBlue droplets injected into SCR systems, it is essential to consider that, under certain conditions, diffusive mechanisms predominate. As emphasized in [18], volume injection is characteristic of configurations in which the spray does not come into contact with the walls of the exhaust manifold—a phenomenon commonly observed in modern automotive applications.

In this context, the vaporization process occurs under isobaric–isothermal conditions, governed by thermal equilibrium with the surrounding environment but significantly influenced by the molecular diffusion of vapors. At the droplet surface, the concentration of urea vapor is higher than in the surrounding medium, creating a radial concentration gradient. According to Fick’s law, this gradient induces a diffusive molar flux *J_az_*, oriented from the droplet surface toward the surrounding gas, and is expressed by the following relation:

(13) $$J_{az}=-D_{AB}\frac{dC}{dr},$$

where *J_az_* [mol/m^2^ s]—the diffusive molar flux, which is a measure of the change in molar mass transported per unit time through the surface unit normal to the direction of travel, *D_AB_* [m^2^/s]—the thermal diffusion coefficient of substance A through substance B, and *dC/dr*—the concentration gradient, where *C* [mol/m^3^]—the concentration, *r* [m]—the radius of the AdBlue droplet.

To calculate the thermal diffusion coefficient, denoted *D_AB_*, the mixture of flue gas and AdBlue vapor is considered. It is known that the vapors have a behavior close to that of ideal gases, the only difference in this case being that the liquid droplets have a negligible volume weight. In addition to these thermophysical properties, the binary diffusion coefficient can influence the vaporization rate, especially for static droplets. The theory describing the diffusion of binary gas mixtures at atmospheric pressure, [7] was developed based on the solution of the Boltzmann equation. Thus, the thermal diffusion equation, according to [19], has the following form:

(14) $$D_{AB}=\frac{1.858\cdot 10^{-3}{T_{g}}^{\frac{3}{2}}{\left(\frac{1}{M_{A}}+\frac{1}{M_{B}}\right)}^{\frac{1}{2}}}{P_{g}\sigma_{AB}^{2}\Omega },$$

where *D_AB_* [m^2^/s]—the thermal diffusion coefficient, *T_g_* [*K*]—the temperature of the gaseous medium in which the diffusion phenomenon takes place, *M_A_*, *M_B_* [g/mol]—the molecular masses of the flue gas and of the AdBlue droplets (*M_A_* = 30.4 g/mol; *M_B_* = 60.05 g/mol), *P_g_* [Pa]—pressure of combustion gases, *Ω*—collision integral, dimensionless, *σ_AB_* [m]—collision diameter. This is given by the following relation:

(15) $$\sigma_{AB}=\frac{\sigma_{A}+\sigma_{B}}{2}.$$

The collision integral *Ω* is a function of temperature and the intermolecular field potential in component *A*, respectively *B* (*σ_A_*—flue gas, *σ_B_*—AdBlue):

(16) $$\Omega =f\left(kT/E_{AB}\right),$$

where *k*—Boltzmann’s constant, *T* [K]—temperature, *E_AB_* [J]—intermolecular interaction energy for the binary system (*E_A_*—combustion gases, *E_B_*—AdBlue). This is in turn determined by the following relation:

(17) $$E_{AB}={\left(E_{A}\cdot E_{B}\right)}^{\frac{1}{2}}.$$

**Figure 3:**
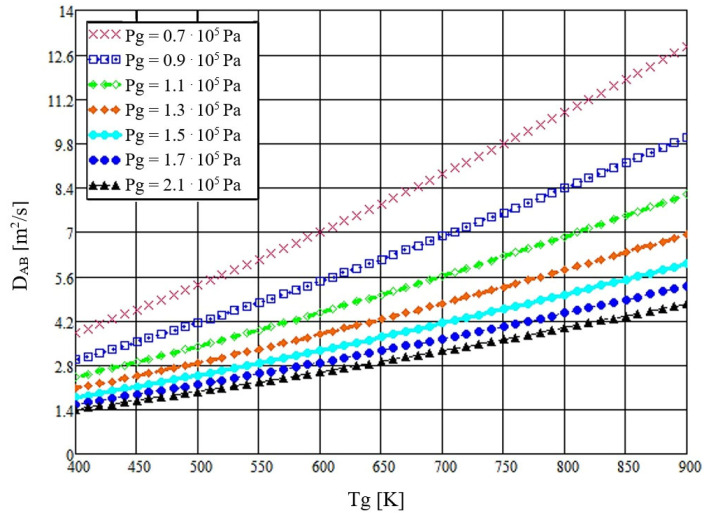
The thermal diffusion coefficient as a function of the temperature of the gaseous medium, taking into account the pressure in the exhaust manifold.

Using the analytical results obtained in MathCad14.0, Figure 3 is plotted with a condensed x-axis (*T_g_* = 400–900 K; tick interval 50 K) to improve readability. The curves show the variation of the diffusion coefficient with exhaust-gas temperature at different exhaust-manifold pressures.

By analysis of the obtained data, it can be observed that we have an increase of the thermal diffusion coefficient with the exhaust gas temperature, and a decrease of it as the pressure increases. The influence of thermal diffusion on the droplet atomization process is significant since, according to the obtained results at temperatures close to 873 K, the coefficient *D_AB_* increases at least 2.6 times with respect to the initial value. The relationship used also takes into account the fact that the exhaust gas pressure undergoes continuous changes due to the transient operating regimes of the diesel engine.

**Figure 4:**
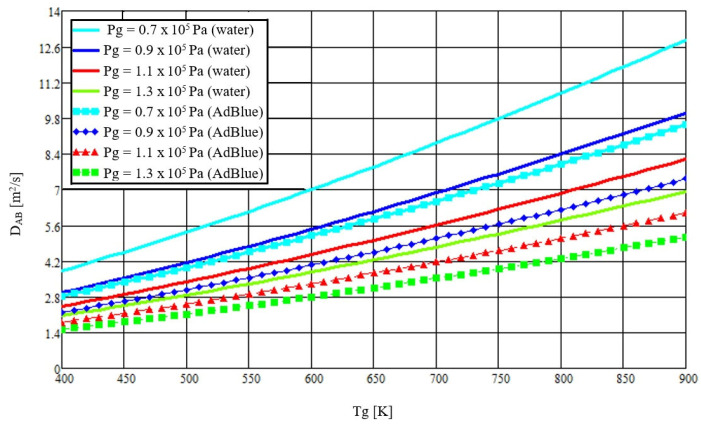
The thermal diffusion coefficient for water and AdBlue as a function of the temperature of the gaseous medium, taking into account the pressure in the exhaust manifold.

To compare the components of the AdBlue solution under identical conditions, Figure 4 is plotted with the same condensed x-axis (*T_g_* = 400–900 K; tick interval 50 K). The thermal diffusion coefficient is shown versus *T_g_* for water and for AdBlue at representative exhaust-manifold pressures.

The changes occurring between the two liquids can be attributed to the different molar masses, since due to higher values in AdBlue droplets, the diffusivity decreases with increasing exhaust gas pressure.

Using the present model developed by the authors, the mass flow rate of AdBlue injected into the catalyst upstream volume can be calculated by taking into account the thermal diffusion process:

(18) $${\dot{m}}_{v}=\frac{dm_{v}}{d\tau }=\frac{d\left(v_{A}M_{A}\right)}{d\tau };{\dot{m}}_{v}=-A_{p}M_{A}D_{AB}\frac{dC}{dr}\Rightarrow {\dot{m}}_{v}=-A_{p}\frac{M_{A}D_{AB}}{RT}\frac{dp_{pv}}{dr},$$

where ${\dot{m}}_{v}$ [kg/s]—the mass flow rate of AdBlue injected into the volume, *A_p_* [m^2^]—the area of the AdBlue droplet, τ [s]—time, ν_A_ [mol]—molar amount of AdBlue, C [mol/m^−3^]—concentration, *r* [m]—radius of the droplet, *p_pv_* [Pa]—partial pressure of the vapor.

If in the expressions for $\frac{dC}{dr}$ and $\frac{dp_{pv}}{\mathrm{dr}}$, $r-r_{p}$ is considered, where, *r*—the radius of the vaporizing droplet and *r_p_*—the radius of droplets considered spherical, then $r-r_{p}\approx r_{p}$, in which case

(19) $${\dot{m}}_{v}=A_{p}M_{A}\frac{D_{\mathrm{AB}}}{r_{p}}\left(C_{s}-C_{r}\right)=A_{p}\frac{M_{A}}{RT_{g}}\frac{D_{AB}}{r_{p}}\left(p_{s}-p_{\rho v}\right).$$

In relation (19), the following notations are used: *s*—saturation state, *p*—droplet, *p_pv_*—vapor partial pressure. Considering the process to be convective, taking into account the *Sh*—Sherwood similarity invariant, it follows that

(20) $${\dot{m}}_{v}=A_{p}M_{A}\frac{Sh\cdot D_{AB}}{d_{PAD}}\left(C_{s}-C_{r}\right),\;{\dot{m}}_{v}=A_{p}M_{A}\frac{Sh\cdot D_{AB}}{d_{PAD}RT_{g}}\left(p_{s}-p_{pv}\right).$$

Substituting the known terms in relation (19) the instantaneous variation of the mass of the AdBlue liquid droplet during the vaporization process in a gaseous medium (diesel engine exhaust gas) is obtained as follows:

(21) $${\dot{m}}_{v}=2\pi d_{\mathrm{mAB}}\frac{M_{A}}{RT}\frac{1.858\cdot 10^{-3}{\left(\frac{1}{M_{A}}+\frac{1}{M_{B}}\right)}^{\frac{1}{2}}}{p\sigma_{AB}^{2}\Omega }\left(p_{S}-p_{\rho w}\right)\frac{1+0.276{\left(\frac{Wd_{PAD}\rho_{AD}}{\eta }\right)}^{\frac{1}{2}}}{{\left(\frac{p\sigma_{AB}^{2}\Omega }{\rho_{AD}\cdot 1.858\cdot 10^{-3}{\left(\frac{1}{M_{A}}+\frac{1}{M_{B}}\right)}^{\frac{1}{2}}}\right)}^{\frac{1}{3}}}$$

where $d_{\mathrm{mAB}}$—average diameter of AdBlue droplet, $\rho_{AD}$—density of AdBlue solution.

**Derivation summary.** Combining the governing balance in (19) with the Sherwood similarity relation (20) yields the working D^2^-law form (21); using the parameter definitions listed below and the transport coefficient from (14) gives the evaporation time in a hot gaseous medium with thermal diffusion, shown in (22). Equation (22) is obtained, which allows the calculation of the diameter of AdBlue droplets injected into a hot gaseous medium, taking into account thermal diffusion:

(22) $$d_{PAD}=\sqrt{d_{\mathrm{mAB}}^{2}-8\cdot \frac{M_{B}}{RT_{g}\rho_{AD}}\cdot D_{AB}\Delta p\left[1+0.276{\left(\frac{ud_{mAD}\rho_{AD}}{\mu_{AD}}\right)}^{\frac{1}{2}}\frac{1}{{\left(\frac{\mu_{AD}}{\rho_{AD}\cdot D_{AB}}\right)}^{\frac{1}{3}}}\right]\tau }.$$

The significance of the quantities in the deduced relation is as follows: *d_PAD_* [m]—the diameter of the liquid droplet undergoing the vaporization process, *R* = 8314.472 [J/molK]—the universal gas constant, *T_g_* [K]—the temperature of the gaseous medium in which the AdBlue droplets are sprayed, *D_AB_* [m^2^/s]—the thermal diffusion coefficient, *u* [m/s]—the velocity of the AdBlue droplet, *ρ_AD_* [kg/m^3^]—the density of the injected liquid (AdBlue), *μ_AD_* [Pa·s]—the dynamic viscosity of the AdBlue solution.

**Figure 5:**
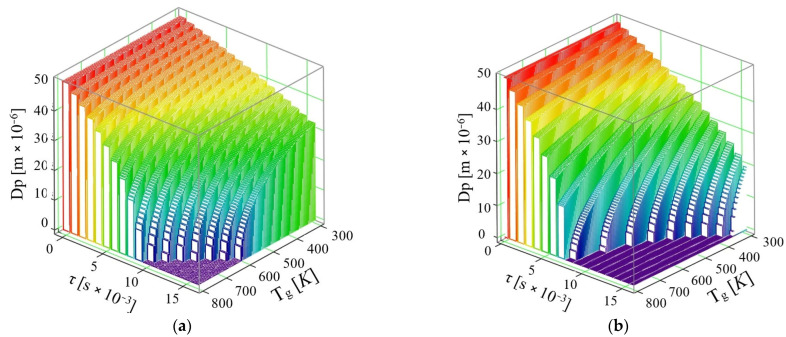
Variation of AdBlue droplet diameter over time with exhaust gas temperature at a velocity *u* = 10 m/s: (**a**) without thermal diffusion and (**b**) with thermal diffusion.

According to studies found in the literature [20,21], a direct connection between the processes of vaporization of substance molecules and convective heat transfer was revealed. On the basis of the relationships deduced above, an equation was derived which allows the determination of the time variation of the diameter of AdBlue droplets injected into a hot gaseous medium. Figure 5 shows the evolution of the AdBlue droplet diameter as a function of the exhaust gas temperature.

For the conducted calculations, an initial temperature of the injected droplets, *T_AD_* = 293 K, was adopted and the density of the AdBlue vapor in the gas stream was considered as *ρ_AD_* = 1087 kg/m^3^. Knowing that the minimum temperature for proper operation of an SCR is 453.15 K, the calculations considered the temperature of the gaseous medium as *T_g_* = 300 ÷ 800 K. The results are illustrated in Figure 5a, where the vaporization of the AdBlue droplet is considered without taking into account the thermal diffusion phenomenon, while in Figure 5b the diffusion coefficient *D_AB_* is introduced, whose value changes as a function of the working fluid and temperature. Interpretation of the cases shows the following:

1. If the phenomenon of thermal diffusion is not taken into account, the calculations show that a drop of AdBlue with diameter *d_PAD_* = 50 × 10^−6^ m at a velocity *u* = 10 m/s can completely vaporize in time τ = 9 × 10^−3^ s at the maximum exhaust gas temperature required for vaporization is *T_g_* = 800 K, while at *T_g_* = 600 K the time required for complete vaporization is τ = 16 × 10^−3^ s. So, the complete vaporization of the AdBlue droplets occurs faster for higher exhaust gas temperatures.
2. If the phenomenon of thermal diffusion is taken into account, at the same initial droplet diameter *d_PAD_* = 50 × 10^−6^ m and the same exhaust gas velocity *u* = 10 m/s, it is found that the AdBlue droplets vaporize much faster than in the previous case (when thermal diffusion is not taken into account). Thus, the vaporization takes place completely in a time τ = 7 × 10^−3^ s at a maximum required flue gas temperature *T_g_* = 767 K, while at temperature *T_g_* = 367 K the minimum required time is τ = 16 × 10^−3^ s.

It is known that the minimum temperature for proper operation of the SCR is 453.15 K [22], in which case it is seen that the diffusive model gives data closer to this value. It is observed, however, that at the considered velocity *u* = 10 m/s in calculus, no values are obtained that attest that the total vaporization of the AdBlue droplets takes place in due time. It can be concluded that the inclusion of the diffusive coefficient is determinant for droplet vaporization.

**Figure 6:**
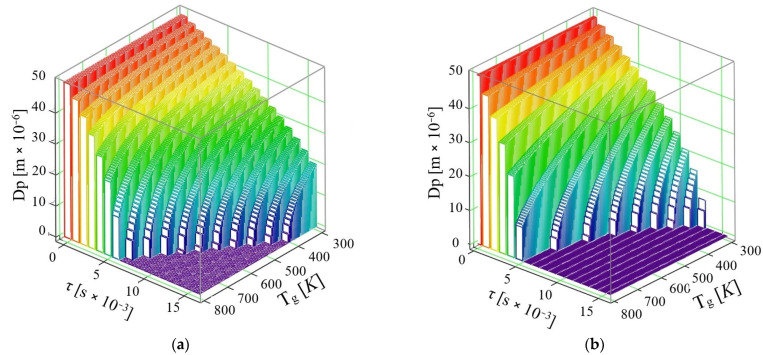
Variation of AdBlue droplet diameter over time with exhaust gas temperature at a velocity *u* = 30 m/s: (**a**) without thermal diffusion and (**b**) with thermal diffusion.

Considering the same AdBlue droplet diameter, *d_PAD_* = 50 × 10^−6^ m, and a velocity of *u* = 30 m/s, Figure 6 is obtained, and it can be seen that an important decrease in the time required for the vaporization process is present.

Interpretation of the data obtained in this case shows the following:

1. If the phenomenon of thermal diffusion is not taken into account, at a velocity *u* = 30 m/s, it can be easily observed that a drop of AdBlue completely vaporizes at the maximum temperature *T_g_* = 800 K in the time τ = 6 × 10^−3^ s, while at temperature *T_g_* = 400 K a vaporization time τ = 16 × 10^−3^ s is required.
2. If the thermal diffusion phenomenon is taken into account, at the velocity *u* = 30 m/s, the vaporization proceeds completely in a time τ = 5 × 10^−3^ s at a maximum flue gas temperature *T_g_* = 800 K, while at temperature *T_g_* = 366 K, the minimum time required for vaporization decreases to τ = 11 × 10^−3^ s. In this model for the gaseous medium velocity *u* = 30 m/s, it is found that already all the droplets are completely vaporized by the end of the travelled path, which is the case corresponding to good functioning of the SCR.

**Figure 7:**
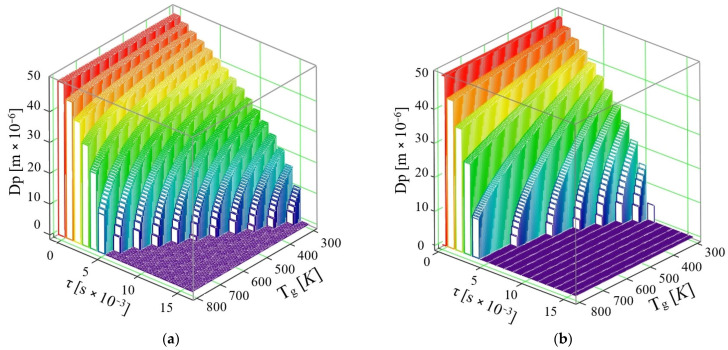
Variation of AdBlue droplet diameter over time with exhaust gas temperature at velocity *u* = 50 m/s: (**a**) without thermal diffusion and (**b**) with thermal diffusion.

Figure 7 shows the resulting data for a maximum AdBlue droplet velocity, *u* = 50 m/s.

As in the previous cases, the two situations are analyzed and the results are as follows:

1. If the phenomenon of thermal diffusion is not taken into account, it can be observed that droplets of AdBlue completely vaporize at a maximum temperature *T_g_* = 800 K, in τ = 5 × 10^−3^ s, and at a temperature *T_g_* = 330 K in τ = 14 × 10^−3^ s.
2. If the phenomenon of thermal diffusion is taken into account, at the velocity *u* = 50 m/s, vaporization proceeds completely in a time τ = 4 × 10^−3^ s at a maximum flue gas temperature *T_g_* = 530 K, while at a temperature *T_g_* = 333 K the time required for complete vaporization is τ = 9 × 10^−3^ s.

By comparing the computational results, it is found that taking thermal diffusion into account is definitive for the volume vaporization of AdBlue droplets moving at different velocities through a hot gaseous medium.

## 4. Considerations on the Vaporization of AdBlue Solution in the Pellicular Regime and the Influence of Ultrasound on This Process

In the film regime, the vaporization process of the AdBlue solution takes place predominantly at the interface between the liquid phase and the gaseous medium, where the droplets come into direct contact with the exhaust gases. The heat transfer at this interface is conditioned by the thermal gradient between the temperature of the flue gas at the interface and that of the injected solution, thus a heat flux transfer to the AdBlue film occurs, followed by the phase transition.

The application of an ultrasonic field—generated through the magnetostrictive effect—at the interface region induces acoustic cavitation, marked by the repeated formation and collapse of vapor microbubbles within the liquid. This process disrupts the film structure of the surface liquid and favors its rupture, resulting in an increase in local turbulence and, consequently, an increase in the heat transfer coefficient. This creates conditions for an accelerated and uniform vaporization (intensification) with a positive impact on the overall efficiency of the NOx catalytic reduction process.

In an experimental investigation carried out by the authors [1], in which fine droplets of AdBlue, generated by injection, were sprayed onto a metal surface at a temperature above the saturation point, an intense vaporization process was observed. Initially, the droplets form a thin film of liquid on the hot surface, but a vapor layer soon develops between the surface and the liquid, leading to a pellicular boiling regime. At this stage, heat transfer is limited compared to nucleic boiling, due to the presence of the vapor layer acting as a thermal insulator.

**Figure 8:**
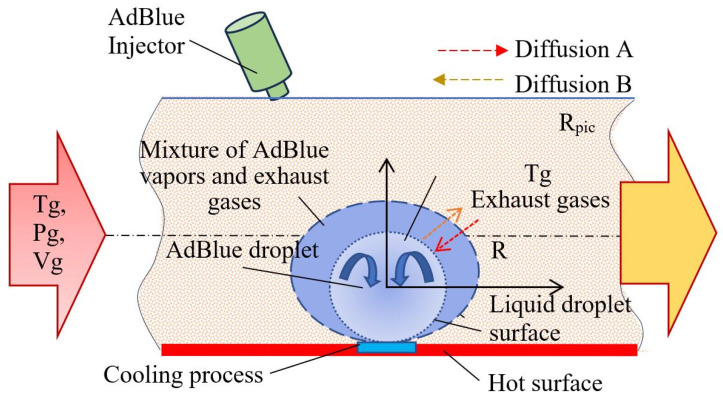
Thermophysical processes of AdBlue droplets vaporizing on a hot surface.

The described phenomenology is summarized in Figure 8, which shows a representative model of the processes occurring during the vaporization of an AdBlue droplet in contact with a superheated surface. This model highlights the sequence of thermal states and the dynamic behavior of the involved phases, providing a solid basis for the further development of a predictive model of ultrasonically assisted heat transfer in the pelt-regime.

In practice, the wall temperature may exceed the saturation values of the vaporizing liquid film. The heat exchange intensity is dictated by the thermal resistance of the vapor film. Since the wall surface has much higher temperatures than the initial temperature value of the AdBlue droplets, over time, overheating of the vapors may occur, and the heat exchange must also take radiation into account. In the first phase of contact of the AdBlue droplets with the hot gas volume, a low-intensity vaporization process takes place due to the short contact time. Most of the droplets end up on the surfaces opposite the AdBlue injector. Once the droplets come into contact with the surface, they suddenly go from an initial low temperature to much higher temperatures in an extremely short time. This leads to the formation of AdBlue vapors, which in a first step separate from the liquid film. The formed vapors mix with the hot gases, which leads to an intensification of the phenomenon by turbulence and heat transfer. Injection of the AdBlue solution on the surfaces leads for a short period of time to a decrease in the local temperature of the exhaust pipe wall, which may lead to a partial reduction of urea decomposition.

For the heat transfer on surfaces, the mass transfer that takes place can be calculated with respect to the AdBlue droplets, determining the vaporization rate, and the variation of droplet diameter with time. It is assumed that the vaporization process occurs when heat transfer takes place between the droplet and the surface with which it comes into contact, and that the droplet temperature is much lower than the temperature of the contact medium. For calculating the mass flow rate variation, the literature [23,24] recommends using Equation (23):

(23) $${\dot{m}}_{ev}=\frac{4\pi \lambda_{v}}{C_{pv}}\frac{d_{PAD}}{2\left(1-\frac{d_{PAD}}{d_{Pi}}\right)}\ln\left[1+\frac{C_{pv}}{l_{v}}\left(T_{S}-T_{PAD}\right)\right],$$

where ${\dot{m}}_{ev}$ [kg/s]—mass flow rate variation of AdBlue droplet, $\lambda_{v}$ [W/mK]—thermal conduction coefficient of AdBlue vapor, *d_PAD_* [m]—diameter of AdBlue droplet during vaporization, *d_P_* [m]—initial diameter of AdBlue droplet, *C_pv_* [J/kgK]—specific heat at constant vapor pressure, $l_{v}$ [J/kg]—latent heat of vaporization, *T_S_* [K]—contact surface temperature, *T_PAD_* [K]—temperature of the AdBlue droplet.

According to the continuity equation, we can express the mass flow rate variation for AdBlue droplets as follows:

(24) $${\dot{m}}_{v}=\rho_{AD}4\pi \frac{d_{PAD}^{2}}{4}u_{p}.$$

Relations (23) and (24) lead to the following expression:

(25) $$\rho_{AD}4\pi \frac{d_{PAD}^{2}}{4}u_{p}=\frac{4\pi \lambda_{v}}{C_{pv}}\frac{d_{PAD}}{2\left(1-\frac{d_{PAD}}{d_{Pi}}\right)}\ln\left[1+\frac{C_{pv}}{l_{v}}\left(T_{S}-T_{PAD}\right)\right].$$

By solving Equation (25), the relation (26) is obtained as below:

(26) $$d_{PAD}=d_{Pi}-\left[\frac{8\lambda_{v}}{C_{pv}\rho_{AD}}\cdot \ln\left[1+\frac{C_{pv}}{l_{v}}\left(T_{S}-T_{PAD}\right)\right]\right]\cdot \tau .$$

In the calculations, the injected droplets are considered to have initial diameter *d_PAD_* = 50 μm before they come into contact with the contact surface. As shown, the vaporization velocity of the AdBlue droplets influences the formation of vapors, which in turn ensures the efficiency of flue gas conversion to NOx in SCR. Depending on the operating regime of the diesel engine, vaporization of AdBlue droplets on hot surfaces (exhaust pipe) can lead to the following situations:

- temperatures below the saturation value of the AdBlue droplets
- AdBlue saturation (boiling) temperatures
- temperatures above boiling temperature when vapors are produced (at which the decomposition of urea in the SCR catalyst takes place)
- superheated temperatures well above saturation when urea can decompose into solid salts and deposit on SCR system components.

**Figure 9:**
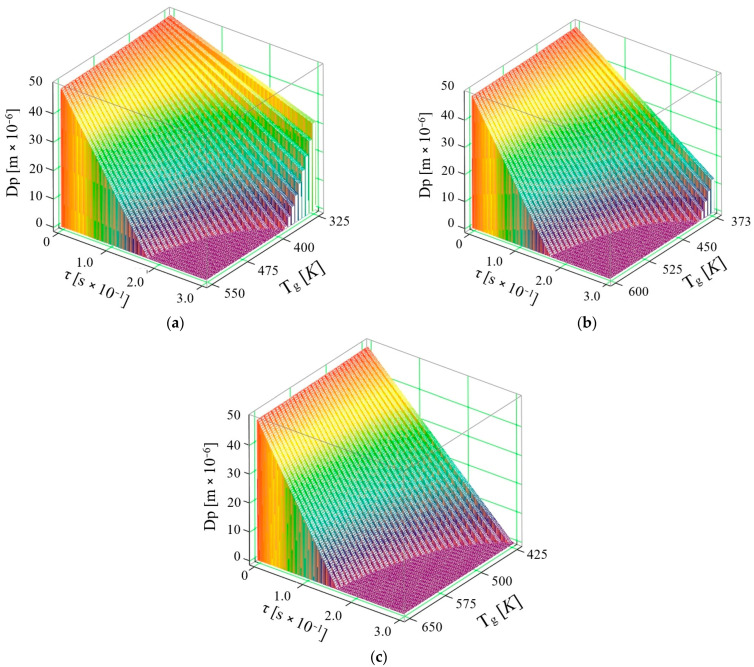
Variation of AdBlue droplet diameter over time with surface temperatures: (**a**) 325–550 K, (**b**) 373–600 K, (**c**) 425–650 K.

Using relation (29), it is possible to calculate the time evolution of the AdBlue droplet diameter when in contact with a hot surface, taking into account the surface temperature. The graphs obtained from the MathCad14.0 calculations are shown in Figure 9.

By analysis of the obtained results, it can be seen that the initial temperature of the surface on which the AdBlue droplets are injected is important because as the temperature increases (as the engine’s thermal regime increases) the vaporization time decreases. Thus, while in the first and second cases the droplets do not fully vaporize, it is found that after the surface temperature reaches 425 K the vaporization process is complete in only 0.3 s. It can be concluded that as the contact surface temperature increases, then the vaporization of AdBlue droplets becomes increasingly pronounced, simultaneously with reaching or even exceeding the saturation temperature. According to the results obtained, in contrast to the case of AdBlue droplets injected into the flue gas volume, the time τ of vaporization increases significantly with surface injection, which can be explained as follows:

- the temperature of the vaporization surface corresponds to the values of the exhaust manifold heated by the exhaust gases, in which case it obviously has lower values than those of the heat source (exhaust gases)
- the exhaust duct is thin-walled, in which case convective heat exchange with the outside environment is favored, leading to rapid cooling, especially at negative outside temperature values
- the heating process of the exhaust pipe is particularly slow when the diesel engine is running cold, a phenomenon which is maintained until the operating temperature is reached
- when vaporizing on surfaces, the droplets injected form a thin film of liquid at high flow rates, in which case this corresponds to a process that no longer involves only AdBlue droplets floating in a hot gaseous medium as in volume vaporization
- the velocity of the droplets moving in a gaseous medium is much higher than in the case of the liquid film flow formed during surface injection
- convective and radiative phenomena can no longer be taken into account in surface vaporization, the phenomenon being rather conductive
- thermal diffusion can be considered as zero since the phenomenon of the interpenetration of the molecules of one body (very fine droplets of AdBlue) between the molecules of another body (exhaust gases) no longer occurs, since the process takes place without the intervention of an external force; when the temperature of the surface onto which the AdBlue jet is injected is close to that of the droplet, the rate of regression of the droplet diameter is attenuated, suggesting that the evaporation process is slower.

It can be easily deduced that the use of methods such as calefaction, which requires the use of a high temperature gaseous medium flowing over strongly atomized droplets by magnetostrictive effect near the solid surface, favors heat exchange and hence increases the conversion efficiency of the SCR catalyst.

## 5. Results and Discussion

### 5.1. Numerical Results and Comparison with the Literature

Using the mathematical models implemented in Mathcad14.0, evaporation parameters for AdBlue droplets were computed. Table 1 reports the absolute evaporation time t together with the D^2^-law normalized value *t*/*d*_0_^2^ for each initial droplet diameter *d*_0_. Because *d*_0_ and flow conditions differ among studies, absolute times are not directly comparable; after size normalization, the values for similar *d*_0_ cluster are within the same order of magnitude, indicating consistency of the present calculations with the published data. The comparatively shorter normalized times obtained under the present conditions are consistent with the ultrasonic actuation and the higher bulk-gas velocities analyzed in Section 2 and Section 4, both of which enhance interfacial heat and mass transfer.

The process of AdBlue droplets vaporization was modeled by different authors as shown in Table 1. Over time, for the vaporization of AdBlue droplets, the models of [27,28] have been established. They compared the diameter of AdBlue droplets after different stagnation times in a hot gaseous medium. Droplet vaporization rate analysis according to a model developed by Lieber et al. [25], showed that an AdBlue droplet with an initial diameter of 10 μm behaves like a regular water droplet, but an AdBlue droplet with a diameter of 8 μm already shows a considerably lower evaporation rate. Through studies, like [25], the sensitivity of the vaporization process as a function of the relative velocity between the droplet and the hot gas was demonstrated.

### 5.2. Discussion of Figures (Trends)

The thermal diffusion coefficient increases monotonically with gas temperature across the pressures evaluated (Figure 3); pressure primarily shifts the curves while preserving the temperature trend. Figure 4 shows analogous behavior for water and AdBlue under identical plotting conditions, with offsets attributable to composition-dependent transport properties. Time-resolved trajectories in Figure 5, Figure 6 and Figure 7 (paired cases without/with thermal diffusion) provide the numerical basis for the vaporization-time entries reported in Table 1. Because initial droplet sizes and boundary conditions differ across studies, absolute times are not directly comparable; therefore, comparisons use D^2^-law normalization *t*/*d*_0_^2^ for like-for-like assessment.

### 5.3. Algorithmic Shortcomings and Limitations

1. Single-droplet scope. An isolated droplet was analyzed; spray-scale interactions (breakup, coalescence, vapor shielding) were not modeled.
2. Near-surface idealization (pellicular regime). Wall micro-roughness, wettability dynamics, and transient wetting transitions were not resolved.
3. Ultrasound representation. Ultrasonic action was incorporated at system level (enhanced atomization/interfacial renewal); the spatio–temporal acoustic/cavitation field was not explicitly solved.
4. Gas-side transport closure. Heat and mass transfer on the gas side used analytical correlations; 3D non-uniformities and turbulence in the exhaust line were not computed.
5. Property ranges. Results relied on temperature/pressure-dependent correlations within the ranges shown; extrapolation beyond these bounds should be done cautiously.
6. Validation breadth. Comparison employed a limited literature set; experiments under matched boundary conditions would further strengthen validation.

## 6. Conclusions

Mathematical models for AdBlue droplet vaporization were formulated and analyzed for both the volumetric (gas-volume) and pellicular (near-surface/film) regimes, with ultrasound represented at system level via the excitation conditions used in the study. The calculations quantify the coupled heat- and mass-transfer processes governing droplet heating and evaporation under exhaust-relevant conditions.

Over the evaluated ranges, the thermal diffusion coefficient increases monotonically with gas temperature, while pressure primarily shifts its magnitude. In the volumetric regime, the evaporation time decreases with increasing gas temperature and relative velocity; this includes the thermal-diffusion term which further shortens the computed evaporation times compared with the no-diffusion variant. In the pellicular regime, higher wall temperatures shorten the time to complete evaporation, whereas conditions near saturation can delay complete vaporization within the available residence/contact time.

As discussed in Section 5, comparison with the published results using D^2^-law normalization (*t*/*d*_0_^2^) shows that the present evaporation times for similar initial diameters cluster within the same order of magnitude as the literature values, supporting the consistency of the calculations with the established trends. Within this context, the analysis indicates that controlling atomization quality, gas temperature/velocity, and wall temperature is important for promoting complete upstream vaporization and for minimizing conditions favorable to deposit formation.

**Scope and limitations.** The study adopted a single-droplet framework (no spray–spray interactions), an idealized near-surface treatment, and a system-level representation of ultrasound (no resolved acoustic/cavitation field). Gas-side heat/mass transfer was closed with analytical correlations rather than 3-D CFD, and validation was limited to comparisons with literature under non-identical boundary conditions.

**Outlook.** Extending the framework to spray-scale interactions, resolved acoustic fields, and coupled SCR kinetics under matched operating conditions would enable tighter uncertainty bounds and a quantitative link to downstream NOx conversion performance.

## Figures and Tables

**Table 1:** Comparison of the vaporization time of droplets in a hot gaseous medium.

Solution	Initial Droplet Diameter	Evaporation Time	Normalized Evaporation Time	Gas Temperature	Gas Velocity	Evaporation Medium	Authors	Relative Error
Unit	[µm]	[s]	[s/mm^2^]	[K]	[m/s]			[%]
Urea solution	750–1240	140	141.41	323	1.1	Hot air	(Lieber et al., 2020) [24]	4.40
	860–1300	18	15.43	523	2.8	Hot air		6.41
	570–1310	10	11.32	673	4.3	Hot air		6.41
	10	0.003	30.00	873	250	Hot air	(Wei et al., 2016) [25]	2.18
	8	0.002	31.25					1.14
	900	21	25.93	473	0	Hot air	(Birkhold et al., 2007) [26]	6.40
	892	8	10.05	673	0	Hot air		6.41
	893	7.5	9.40	773	0	Hot air		2.80
	70	0.78	159.18	673	0	Hot air	(Kontin et al., 2010) [27]	6.53
		0.04	8.16	900	100	Hot air		3.20
AdBlue	50	0.016	6.40	600	10	Exhaust gases	Present study	0
		0.009	3.60	800				10.29
		0.016	6.40	367				0
		0.007	2.80	767				0
		0.005	2.00	800	30	Exhaust gases		8.00
		0.011	4.40	366				0
		0.016	6.40	400				0

## Data Availability

Some or all data, models, or code generated or used during the study are available from the corresponding author by request.
